# Expression signatures of HOX cluster genes in cervical cancer pathogenesis: Impact of human papillomavirus type 16 oncoprotein E7

**DOI:** 10.18632/oncotarget.16619

**Published:** 2017-03-28

**Authors:** Sweta Sharma Saha, Rahul Roy Chowdhury, Nidhu Ranjan Mondal, Sudipta Roy, Sharmila Sengupta

**Affiliations:** ^1^ National Institute of Biomedical Genomics, Kolkata, West Bengal, India; ^2^ Department of Gynecology, Saroj Gupta Cancer Centre and Research Institute, Kolkata, West Bengal, India; ^3^ Sri Aurobindo Seva Kendra, Kolkata, West Bengal, India

**Keywords:** cervical cancer, human papillomavirus, HOX genes, E7, chromatin marks

## Abstract

The Homeobox (HOX) genes encode important transcription factors showing deregulated expression in several cancers. However, their role in cervical cancer pathogenesis, remains largely unexplored. Herein, we studied their association with Human Papillomavirus type 16 (HPV16) mediated cervical cancers. Our previously published gene expression microarray data revealed a significant alteration of 12 out of 39 HOX cluster members among cervical cancer cases, in comparison to the histopathologically normal controls. Of these, we validated seven (HOXA10, HOXA13, HOXB13, HOXC8, HOXC9, HOXC11 and HOXD10) by quantitative real-time PCR. We identified decreased HOXA10 expression as opposed to the increased expression of the rest. Such decrease was independent of the integration status of HPV16 genome, but correlated negatively with E7 expression in clinical samples, that was confirmed *in vitro*. HOXA10 and HOXB13 revealed association with Epithelial-Mesenchymal Transition (EMT). While HOXA10 expression correlated positively with E-Cadherin and negatively with Vimentin expression, HOXB13 showed the reverse trend. Chromatin immunoprecipitation study *in vitro* revealed the ability of E7 to increase HOX gene expression by epigenetic regulation, affecting the H3K4me3 and H3K27me3 status of their promoters, resulting from a loss of PRC2-LSD1 complex activity. Thus, besides identifying the deregulated expression of HOX cluster members in HPV16 positive cervical cancer and their association with EMT, our study highlighted the mechanism of HPV16 E7-mediated epigenetic regulation of HOX genes in such cancers, potentially serving as bedrock for functional studies in the future.

## INTRODUCTION

Cervical cancer is the fourth leading cancer among women globally with HPV16 infection alone associating with ~50% of the cases [1–5]. HPV16 E7 is one of the driver molecules responsible for HPV-induced cellular transformation [6]. The ability of HPV16 E7 to interact with the host-encoded proteins has been well-documented [7–8]. Such functional interactions, specifically with transcription factors, cumulatively influence the transcriptional process and global gene expression creating an environment conducive to HPV-mediated cervical cancer pathogenesis. However, in a recent report from our laboratory [9] we proposed a mechanism of E7-mediated increase of gene expression globally, by affecting lncRNA HOTAIR expression and function. Such functional inactivation of HOTAIR, in turn, could impair Polycomb Repressive Complex 2 (PRC2) and Lysine Specific Demethylase 1(LSD1) recruitment resulting in concomitant loss of global gene silencing H3K27me3 marks and gain of gene activating H3K4me3 marks, respectively. However, such proposition needs experimental validation.

HOX genes are a family of transcription factors that show deregulated expression in a variety of cancers and known as targets of PRC2-complex during embryonic development [10–11]. The class I HOX genes comprise of 39 members distributed across four clusters localized in four different chromosomes - HOXA at 7p15.3, HOXB at 17p21.3, HOXC at 12q13.3, and HOXD at 2q31[12–14]. Although many of the downstream targets of HOX genes are still not fully defined, it has been shown that HOX genes are integral to normal temporo-spatial limb [15] and organ [16] development along the anterior-posterior (A-P) axis.

Since HOX cluster members function as transcription factors, the common thought was that these show enhanced expression in cancers driving carcinogenesis by activating the downstream signalling cascades. However, several studies reported reduced expression of HOX cluster encoded molecules. Aberrant expression of HOX cluster genes is integral to a network of regulatory mechanisms involved in normal adult tissue homeostasis, and maintenance and activation of stem cell self-renewal process, crucial to malignant transformation [17–20]. The expression of genes belonging to the HOXC family, particularly HOXC4 to HOXC9, HOXC11, and HOXC13, shows predominant increase in most of the solid tumour types, except ovarian cancers. The two HOX genes most commonly activated in solid tumours are HOXA9 and HOXB13 [21]. Such activation of HOX cluster members are closely linked to the process of Epithelial-Mesenchymal Transition (EMT) [22–24]. However, studies on expression profiling of HOX cluster genes in cervical cancers are mostly based on cell lines [25–28], with only a couple of studies employing global gene expression profiling of cervical tissues [29–30]. However, none of these studies on cervical cancer cell lines or tissues, have taken into account the presence of HPV or expression of viral oncogenes. McLaughlin-Drubin et al., in 2011 [31], highlighted the increased expression of the PRC2-target HOX cluster genes in the presence of E7. This is concomitant with global loss of chromatin suppressive H3K27me3, despite the increased EZH2 expression, under the influence of E7 [31]. However, the impact of E7 on the gene activating H3K4me3 and gene silencing H3K27me3 marks, specifically at the HOX promoters, has not been studied thus far.

Therefore, in this study, we focussed on elucidating the expression profile of HOX cluster member genes associated with cervical cancer pathogenesis, and their probable association with the Epithelial Mesenchymal Transition (EMT). We also aimed at identifying the association of HOX cluster expression with viral factors like HPV16 integration status and E7 expression among the cervical cancer cases. Finally, we elucidated the role of HPV16 E7 in epigenetically affecting the expression of HOX cluster genes through concomitant functional abrogation of PRC2-LSD1 complex in cervical cancer cell lines. Towards this, we estimated the H3K4me3 and H3K27me3 status of the promoters of the HOX genes with altered expression.

## RESULTS

### HOX cluster genes show significant expression deregulation among cervical cancer samples

To identify the HOX cluster members with deregulated expression among HPV16 positive cervical cancer cases in comparison to the histopathologically normal control samples (HPV negative and HPV16 positive), microarray data generated on 20 HPV16 positive cervical cancer cases, 11 HPV16 positive histopathologically normal samples and 11 HPV negative histopathologically normal samples (Accession No: GSE67522) was used. The histopathologically normal samples were grouped together for comparison, as the HPV negative and HPV16 positive normal samples did not reveal any differentially expressed genes as described in our previous study [9].

**Figure 1:**
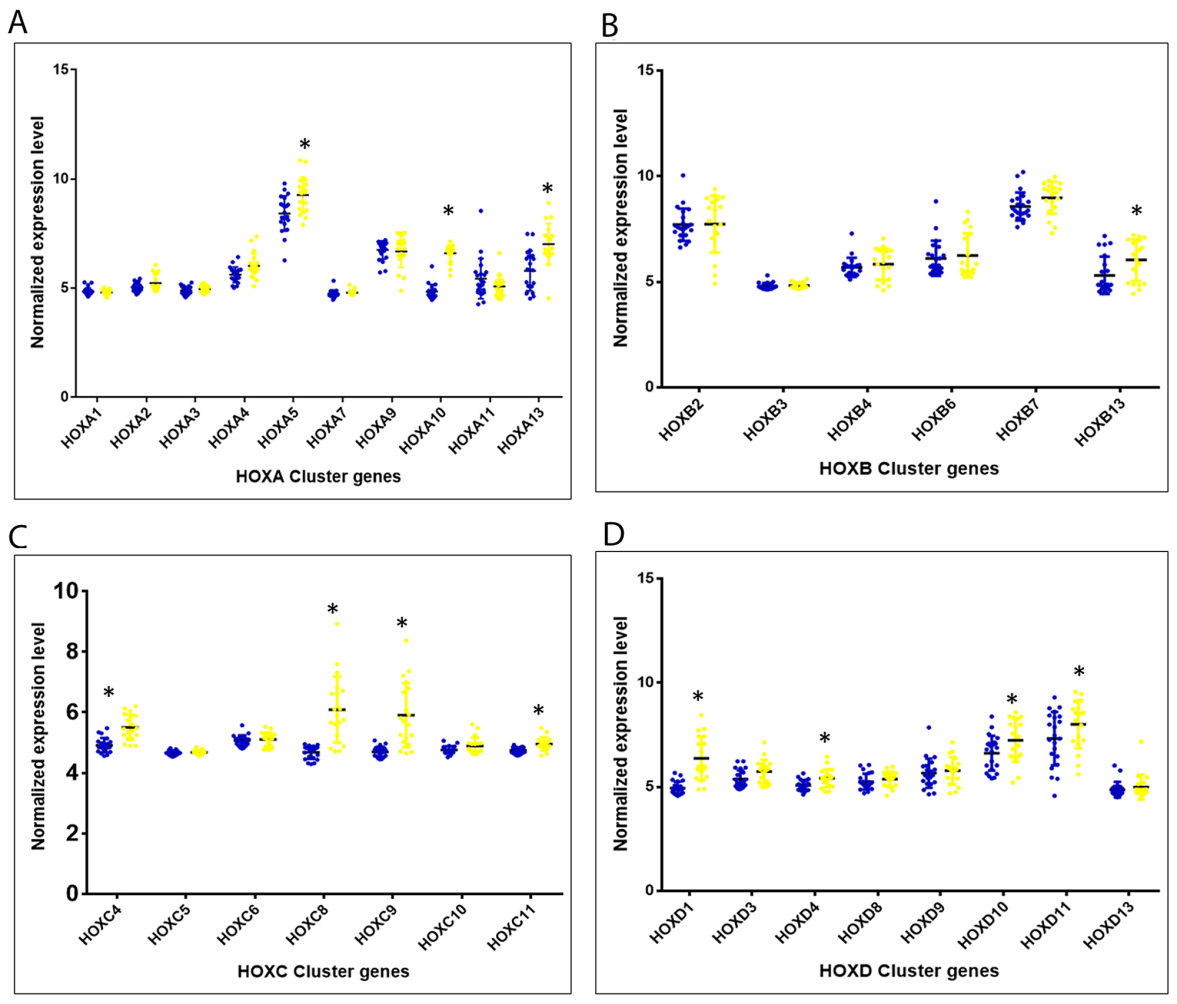
Altered expression of 4 HOX clusters (covered in microarray) among cervical cancers as compared to histopathologically normal samples **(A – D)** represent genes belonging to the clusters, HOXA, HOXB, HOXC and HOXD, respectively. (Blue dots: Histopathologically normal controls samples; Yellow dots: HPV16 positive cervical cancer cases; * Statistically significantly up-regulated among cervical cancer cases with p<0.05)

Out of 39 HOX cluster genes, the microarray analysis revealed expression levels of 31 genes. Of these, 12 members of the HOX cluster portrayed significant differential expression among the cervical cancer cases, as compared to the histopathologically normal controls (Figure 1; Supplementary Table 1).

.

### Validation of expression changes of seven deregulated HOX cluster members

**Figure 2:**
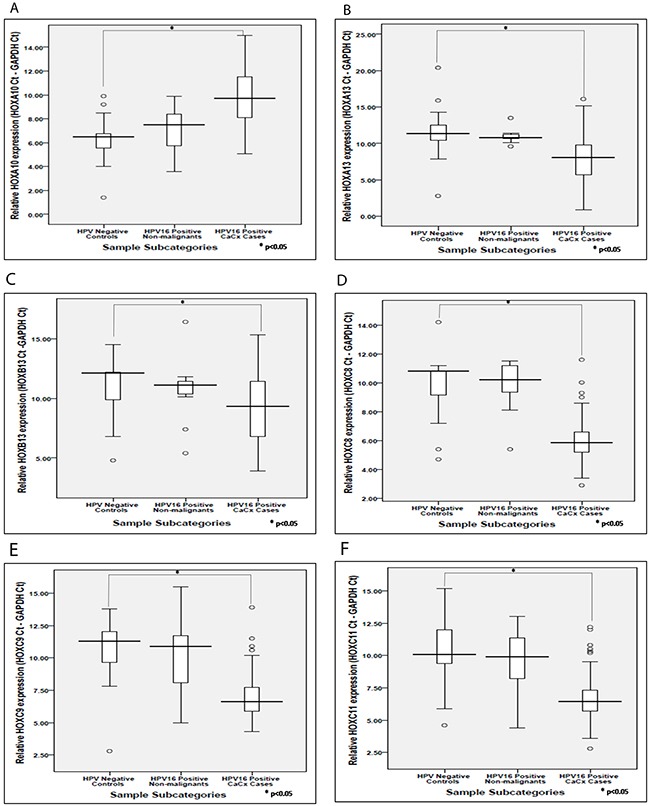
Box plots representing distribution of the expression levels of the HOX cluster genes based on real time PCR analysis **(A – F)** corresponds to HOX cluster members such as HOXA10, HOXA13, HOXB13, HOXC8, HOXC9, HOXC11, respectively, among different categories of cervical samples.

We validated the expression profile of seven out of the twelve deregulated HOX cluster members, HOXA10, HOXA13, HOXB13, HOXC8, HOXC9, HOXC11 and HOXD10, using SYBR green based quantitative real-time PCR. For such analysis, we used a sample set of seventy cervical cancer cases (44 samples harbouring episomal HPV16 and 26 samples harbouring integrated HPV16 genomes) and thirty histopathologically normal cervical samples (11 HPV16 positive and 19 HPV negative). Such analysis, confirmed significant altered expression of all seven HOX cluster members among the cervical cancer cases, in comparison with the HPV negative control samples (Supplementary Table 2). Of these, upregulation of HOXD10 has been previously reported from our laboratory [9]. HOXA10 was the only member showing significantly reduced expression, as opposed to the rest revealing significant increase in expression (Figure 2). However, none of these HOX cluster members showed differential expression between HPV16 positive non-malignant samples and HPV negative control samples, which was concordant with our observations using microarray analysis. The cervical cancer cases harbouring episomal and integrated HPV16 also failed to show significant differences in the expression levels of all the seven HOX cluster genes. This was indicative of the fact that the expression of HOX cluster genes was independent of the physical status of the HPV16 genome.

### Expression of HOXA10 and HOXB13 correlates with expression of EMT markers, E-Cadherin and Vimentin

Deregulated expression of several HOX genes in cervical cancer cases prompted us to interrogate the impact of such changes in cervical cancer pathogenesis. Existing reports [21–24], highlight the role of some HOX cluster members in driving metastasis, by the induction of Epithelial Mesenchymal Transition (EMT). We therefore determined the expression of EMT markers, E-Cadherin and Vimentin, by quantitative real-time PCR, among the same set of samples used for the validation of the expression levels of the HOX cluster members.

**Figure 3:**
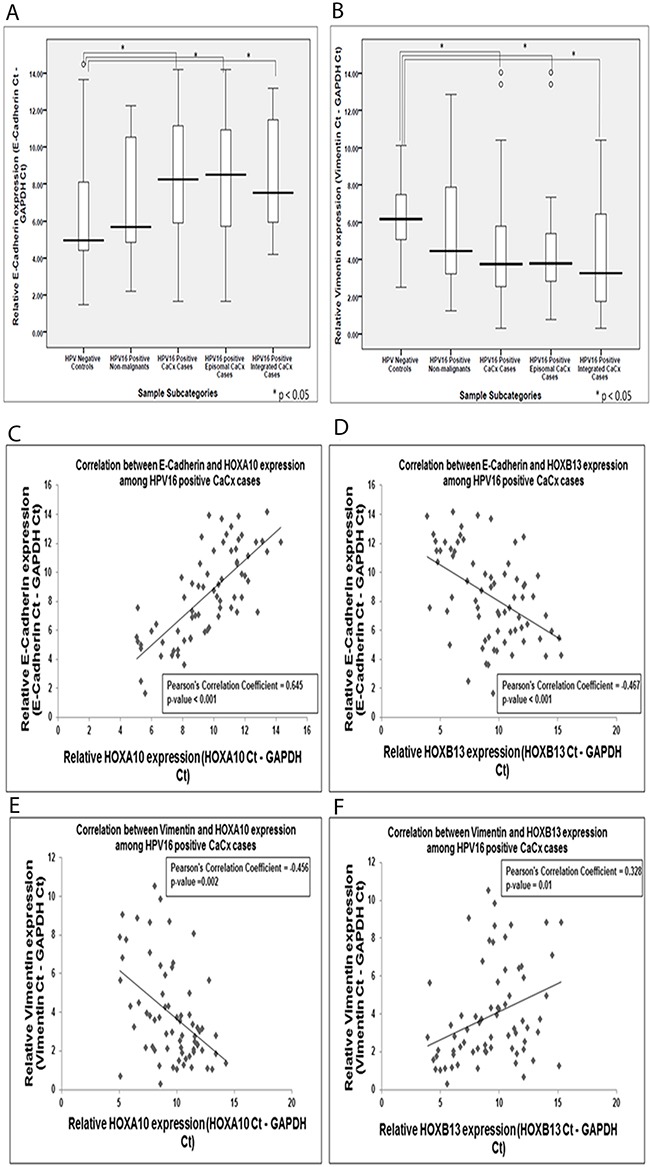
Box plots representing expression levels of the E-Cadherin and Vimentin among the different categories of cervical samples and their correlation with HOXA10 and HOXB13 expression, among cervical cancers **(A)** Relative expression of E-Cadherin (E-Cadherin Ct – GAPDH Ct) **(B)** Relative expression of Vimentin (Vimentin Ct – GAPDH Ct) of samples **(C)** Correlation plot depicting significant positive correlation between HOXA10 and E-Cadherin expression **(D)** Correlation plot depicting significant negative correlation between HOXB13 and E-Cadherin expression **(E)** Correlation plot depicting significant positive correlation between HOXA10 and Vimentin expression **(F)** Correlation plot depicting significant negative correlation between HOXB13 and Vimentin expression.

E-Cadherin expression was significantly decreased among the cervical cancer cases (9.85-fold; p=0.024) as compared to the HPV negative control samples. The cervical cancer samples with episomal HPV16 genome showed a reduction by 11.79-fold (p=0.03), while the cervical cancer samples with integrated HPV16 genomes showed a reduction by 6.02-fold (p=0.04) (Figure 3A). However, such difference between the two cervical cancer subtypes was not statistically significant. Vimentin, on the other hand, showed a significantly increased expression among the cervical cancer cases (3.83-fold; p=0.001), as compared to the HPV negative control samples. The cervical cancer samples with episomal and integrated HPV16 genomes showed significantly increased expression of Vimentin by 3.66-fold (p<0.001) and 5.44-fold (p=0.014), respectively (Figure 3B). Both E-Cadherin and Vimentin expression failed to differ significantly between the cervical cancer cases with episomal and integrated HPV16 genomes.

Expression of both the EMT markers, E-Cadherin and Vimentin, showed correlation with the expression of the HOX cluster members, HOXA10 and HOXB13 (Supplementary Tables 3 and 4). While HOXA10 showed a positive correlation with E-Cadherin (Pearson's Correlation Coefficient=0.645; p=0.0017) and a negative correlation with Vimentin (Pearson's Correlation Coefficient=-0.456; p=0.014), the reverse trend was observed for HOXB13. HOXB13 showed a negative correlation with E-Cadherin (Pearson's Correlation Coefficient= -0.467; p=0.0014) and a positive correlation with Vimentin (Pearson's Correlation Coefficient=0.328; p=0.035). Thus the correlation plots of HOXA10 and HOXB13 with the EMT markers (Figure 3C–3F), suggest a role of HOXA10 and HOXB13 in driving metastasis in cervical cancer.

### HOXA10 and HOXD10 expression correlates with HPV16 E7 expression

**Figure 4:**
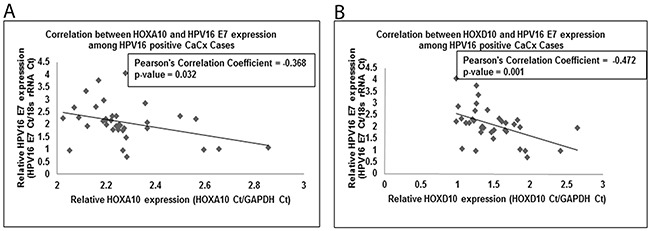
Linear regression analysis for correlation of **(A)** HOXA10 and **(B)** HOXD10, with HPV16 E7 expression among cervical cancers

Existing reports show enhanced E-Cadherin expression in response to silencing of E7 in HPV16 positive keratinocytes [32]. Thus, the correlation between HOXA10 and HOXB13 and the EMT markers, E-Cadherin and Vimentin, was indicative of an interactive role of E7 and HOX cluster genes. In addition, HPV16 E7 impairs the activity of the Polycomb repressive complex, a known regulator of HOX cluster genes, resulting in decreased global H3K27me3 marks [31]. Such observations prompted us to determine if the expression of HOX cluster genes showed any correlation with HPV16 E7 expression among the case samples. For this, we first considered the microarray based data for the HOX genes showing deregulated expression (Supplementary Table 5). Subsequently, we validated the results employing qRT-PCR data on larger number of samples. Only, HOXA10 and HOXD10 expression showed a statistically significant negative correlation with HPV16 E7 expression (Pearson's correlation coefficients=-0.368 and -0.472, respectively; p=0.032 and 0.001, respectively; Figure 4). We further confirmed the negative correlation between HOXA10 and HPV16 E7 expressions *in vitro*, where HOXA10 showed significantly reduced expression (450-fold, p<0.001) in C33A cells transfected with E7 expressing plasmid, as compared to untransfected C33A cells (Supplementary Figure 1).

The increased HOXD10 expression among the cervical cancer cases recorded in our previous report, correlated negatively with lncRNA HOTAIR expression [9]. HOTAIR is known to *trans* regulate HOXD10, through PRC2-LSD1 complex recruitment. However, the reduction of HOTAIR expression among the cervical cancers could be responsible for the loss of such gene silencing and hence HOXD10 upregulation, which demanded further validation.

### HPV16 E7 mediated alteration of HOX gene expression and histone methylation (H3K4me3 and H3K27me3) status at the promoters of HOX cluster members

**Figure 5:**
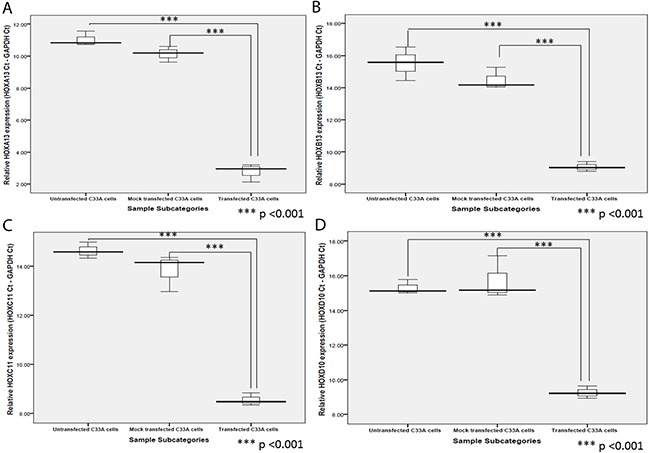
Box plots representing expression of **(A)** HOXA13, **(B)** HOXB13, **(C)** HOXC11, and **(D)** HOXD10, in C33A cells post-transfection of pcDNA3.1-HPV16 E7 vector in comparison to the untransfected and mock (empty vector) transfected C33A cells

In our previous study, the increased expression of a large number of genes in cervical cancer cases, was explained through probable abrogation of lncRNA HOTAIR function in PRC2-LSD1 complex recruitment and repression of gene expression, under the influence of E7 [9]. Thus, we hypothesized that HPV16 E7 might have an important role to play in the activation of HOX cluster genes through abrogating PRC2-LSD1 complex activity, despite the fact that some of the HOX cluster members failed to show any significant correlation with HPV16 E7. We therefore aimed to identify the expression levels and status of gene activating (H3K4me3) and gene silencing (H3K27me3) chromatin marks at the promoters of four HOX cluster genes (HOXA13, HOXB13, HOXC11 and HOXD10). We selected these genes in view of their significantly enhanced expression among cervical cancer cases (Supplementary Tables 1 and 2). We observed that all the four members showed increased expression in the HPV negative cervical cancer cell line, C33A, upon transfection with HPV16 E7 expression plasmid (Figure 5; Supplementary Table 6).

**Figure 6:**
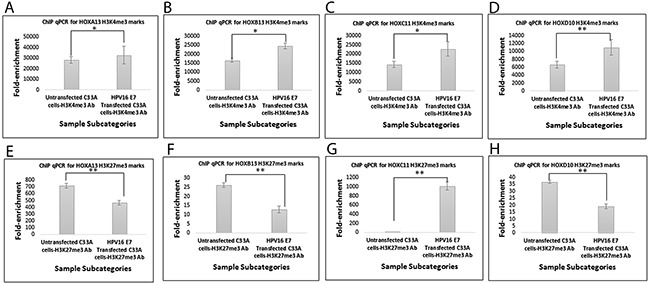
q-PCR based analysis of enrichment of H3K4me3 and H3K27me3 marks at the promoters of HOX cluster genesin HPV negative and E7 expressing C33A cells Fold-enrichment of H3K4me3 marks at **(A)** HOXA13, **(B)** HOXB13, **(C)** HOXC11, **(D)** HOXD10 promoters, Fold-enrichment of H3K27me3 marks at **(E)** HOXA13, **(F)** HOXB13, **(G)** HOXC11, **(H)** HOXD10 promoters. (* p<0.05; **p<0.01; Abbreviations: Ab=Antibody).

The enhanced expression of the four HOX cluster members was concomitant with significant alterations in the gene activating H3K4me3 marks and gene silencing H3K27me3 marks, at their promoter regions (Supplementary Table 7). The promoter regions of all four HOX cluster members, showed an enrichment of H3K4me3 marks with a reduction in H3K27me3 marks in three of the members, HOXA13, HOXB13 and HOXD10 (Figure 6), explaining the increased expression of HOX cluster members. HOXC11 showed a concomitant enrichment of both H3K4me3 and H3K27me3 marks, but a higher fold-enrichment for H3K4me3 marks as compared to H3K27me3 marks, indicating progression towards gene activation. Such observations clearly establish our model of E7-dependent epigenetic regulation of HOX genes, through abrogation of PRC2-LSD1 complex activity.

## DISCUSSION

The role of HOX gene deregulation in cancer development remains unexplored not only in cervical cancers but in other cancers as well. Thus, we undertook this study to identify the HOX cluster members with altered expression, specifically in HPV16 positive cervical cancers. We observed significantly increased expression of several HOX cluster members, excepting HOXA10, among the cervical cancer cases, in comparison to the healthy controls. This observation impressed upon us, the possibility that HOX gene expression increase could in turn bring about the activation of several cancer-related pathways, during cervical cancer development. None of the HOX cluster genes with altered expression levels, showed any association with the integration status of the viral genome (episomal/integrated). However, HPV16 E7 seemed to play an important role in the activation of HOX cluster genes despite the fact that some of the HOX cluster members failed to show any significant correlation with HPV16 E7.

The HOX cluster members that revealed altered expression in cervical cancer cases in our study, also show deregulated expression in one or more of the other cancer types. Similar to our observations, HOXA13 shows enhanced expression in oesophageal squamous cell carcinoma, gastric cancer and hepatocellular carcinoma [33–35]. However, its mode of action in cancer development is still unclear. HOXB13 activation, on the other hand, has been shown to be associated with metastasis through activation of MMP-9 [23, 36–37], in a variety of solid tumours. The existence of a similar mechanism in cervical cancer demands further investigations. Studies on HOXC cluster members have also identified increased expression of HOXC8, HOXC9 and HOXC11, as observed by us in this study. HOXC8 correlates negatively with tumour growth [37] among pancreatic ductal adenocarcinomas, while showing increased expression in cervical cancers [38]. Activation of HOXC9 and HOXC11 in various cancers has been associated with cell proliferation and invasion [39–41]. A few studies have also highlighted that HOX cluster members drive metastasis, specifically by inducing Epithelial Mesenchymal Transition (EMT) [22–24]. In our study, the decreased HOXA10 expression and increased HOXB13 expression were associated with reduced E-Cadherin and enhanced Vimentin expression, proposing the need for further studies to establish HOXA10 and HOXB13 as biomarkers of metastasis in cervical cancer.

The increased expression of majority of the HOX cluster members was also in line with our hypothesis of HPV16 E7 driven abrogation of PRC2-LSD1 complex activity leading to loss of gene silencing. A significant correlation of HOXA10 and HOXD10 expression with HPV16 E7 expression highlighted the impact of E7 on HOX gene expression. One of the possible mechanism of HOXA10 reduction could be through HPV16 E7-dependent activation of DNMT1 leading to HOXA10 promoter methylation [22, 41]. On the other hand, in our earlier report, we interpreted the increased HOXD10 expression in cervical cancer cases to be the result of E7-dependent inactivation of HOTAIR mediated gene silencing through PRC2-LSD1 complex [9].

Although E7 expression did not show significant correlation with the expression of majority of the HOX cluster members among the cervical cancer samples, E7 overexpression in HPV negative cervical cancer cell line resulted in the activation of HOX cluster genes. Such differences in clinical and *in vitro* observations could be due to the heterogeneity of cancer tissues as opposed to the homogenous pool of cells *in vitro*. The gain of H3K4me3 and loss of H3K27me3 marks at the promoters of HOX cluster genes could explain the activated expression of such genes, under the influence of HPV16 E7 expression. McLaughlin-Drubin et al., [31] had earlier shown that E7 expression reduces global H3K27me3 marks and increases the expression of PRC2-target HOX genes in Human Foreskin Keratinocytes. They interpreted this to be the result of transcriptional induction of KDM6A and KDM6B H3K27 specific demethylases, despite increased expression of EZH2 under the influence of E7 that results in the loss of EZH2 activity through Akt pathway mediated phosphorylation of EZH2 [31]. In this study, we clearly observed the concomitant and significant enrichment of H3K4me3 and reduction of H3K27me3 marks at the HOX cluster promoters, despite increased expression of the PRC2 complex member EZH2 as recorded in our previous study (9). Thus taken together, through this study we establish the mechanism of E7-mediated activation of HOX gene expression through functional abrogation of HOTAIR, ultimately suppressing the PRC2-LSD1 complex activity, a model that we proposed in our previous study [9].

In conclusion, along with identifying altered expression of HOX cluster genes and their association with metastasis in HPV16 related cervical cancers, our study also highlighted the master regulatory role of HPV16 E7 in modulating the expression of HOX cluster genes. Such expression alteration predominantly involved enrichment of H3K4me3 and loss of H3K27me3 marks at the gene promoters. Thus global studies employing ChIP-Seq for identifying the status of H3K4me3 and H3K27me3 marks at the gene promoters, along with RNA-Seq, seem to be important to identify the concomitant changes in the transcriptome brought about by HPV16 E7. This would further identify and confirm the role of HPV16 E7 in the epigenetic regulation of host cellular transcription. Such studies would also help in identifying molecular targets for therapy of HPV-active cervical cancer, which have distinctly different molecular phenotypes from HPV-inactive cervical cancer, as has been identified in a recent report studying TCGA cervical cancer data [42].

## MATERIALS AND METHODS

### Clinical samples and HPV16 Status

The healthy control cervical biopsy samples were derived from married women aged 36 – 70 years (median: 52 years) without any prior history of cervical dysplasia/malignancy, from Calcutta Medical College and Hospital, Kolkata, West Bengal, India and were histopathologically confirmed as normal. The Cervical cancer samples used for this study were derived from married subjects aged 34 – 65 years (median: 52 years) attending a cancer referral hospital (Saroj Gupta Cancer Centre and Research Institute, South 24 Parganas, West Bengal, India). The cervical cancer biopsy samples were histopathologically confirmed as invasive squamous cell carcinomas (majority of which were diagnosed as moderately differentiated squamous cell carcinoma pathologically) categorised using the WHO classification and clinically diagnosed as stage III and above as per FIGO classification. Samples included in the different study groups did not differ significantly in terms of their median age.

The samples were collected with informed consent from the participants, approved by the ethical committee for human experimentation of the National Institute of Biomedical Genomics (NIBMG) and frozen post collection. We performed all experiments in accordance with the approved ethical guidelines and regulations. All such clinical samples were tested for their HPV/HPV16 status by PCR based detection as described earlier [9]. Confirmation of the integration or episomal status of HPV16 was done employing APOT-coupled Taqman assay as described previously [43].

### Microarray expression analysis of HOX cluster members

The microarray data was generated using Illumina HumanHT*-*12 v4 Expression Bead Chip array based platform generated by our laboratory [9] and submitted to the GEO repository (Accession Number: GSE67522, http://www.ncbi.nlm.nih.gov/geo/query/acc.cgi?acc=GSE67522). This data was used to identify the distinct differences of HOX cluster members at the gene expression level, between the HPV16 positive cervical cancer cases and the histopathologically normal control samples.

### RNA isolation, reverse transcription and quantitative real-time PCR

RNA isolation, reverse transcription and quantitative real-time PCRs were all performed, as described previously [9]. The primers used for the expression analysis using qRT-PCR are listed in Supplementary Table 8.

### Cell culture and transfection

HPV negative cell line C33A and HPV16 positive cell line SiHa were cultured in DMEM supplemented with 10% FBS, 50 Units/ml of penicillin and 50 μg/ml of streptomycin at 37°C and 5% CO_2_. C33A cells were transfected with1 μg of plasmid pcDNA3.1-HPV16 E7 vector generated in our laboratory [9], using Lipofectamine 2000 reagent according to manufacturer's protocol. The cells were harvested and washed with 1X PBS, pH 7.4, trypsinized and collected by centrifugation at 300 xg for 10 minutes. The transfected cells were used for RNA and protein isolation. The expression of HPV16 E7 was confirmed by Western blot (Supplementary Figure 2; as shown in Ref. 9) The transfection experiments were carried out in three sets, each in triplicates.

### Chromatin immunoprecipitation and quantitative real-time PCR

SiHa and C33A cells were cultured as described above. For the analysis of H3K4me3 and H3K27me3 marks, we used SiHa cells and C33A cells, untransfected or transfected (HPV16 E7 expressing). The cells were cross-linked using formaldehyde at 1% final concentration and incubated at room temperature for 10 mins. The cross-linking was quenched using glycine at a final concentration of 0.125M and incubated at room temperature for 5 mins, followed by washing twice with 1X PBS. The cells were harvested by scraping in 1X PBS (with Protease Inhibitor cocktail) and pelleting the cells by centrifugation at 300xg for 5 mins, at 4°C. The cells were re-suspended in 1ml of SDS lysis buffer (1% SDS, 10mM EDTA, 50mM Tris-HCl pH 8.1 and protease inhibitors before use) and incubated on ice for 15 mins. The lysate was sonicated at 30% amplitude, 14 × 10 secs pulses, 20 secs pauses, followed by centrifugation at 14,000 rpm at 4°C. The lysate was pre-cleared with 80μl of 50% slurry of Protein A Agarose beads for 60 mins at 4°C, followed by centrifugation at 4,000 rpm for 1 min at 4°C. Specific antibody (5μg) was added to the supernatant and precipitated overnight at 4°C with rotation. After overnight rotation, 60μl of blocked Protein A Agarose beads (1ml 50% bead slurry, 0.5mg BSA) were added to the antibody/protein complex, followed by rotation for 60 mins at 4°C. The beads were pelleted by centrifugation at 1,000 rpm for 1 min at 4°C, followed by sequential washing with 1X Low Salt Immune Complex wash buffer (0.1% SDS, 1% TritonX-100, 2mM EDTA, 20mM Tris-HCl pH 8.1, 150mM NaCl), 1X High Salt Immune Complex wash Buffer (0.1% SDS, 1% TritonX-100, 2mM EDTA, 20mM Tris-HCl pH 8.1, 500mM NaCl), 1X LiCl Immune Complex wash Buffer (0.25M LiCl, 1% NP-40, 1% Deoxycholic acid sodium salt, 1mM EDTA, 10mM Tris-HCl pH 8.1) and 1X TE (10mM Tris pH 8.1, 1mM EDTA). The beads were eluted with freshly prepared elution buffer (1% SDS, 0.1M NaHCO_3_) and incubated at room temperature for 30 mins with agitation. The DNA was reverse cross-linked by adding 12μl of 5M NaCl per 100μl elute and incubated at 65°C overnight, followed by RNase A treatment and purified using PCR purification kit.

The purified DNA was then used for SYBR green based qPCR estimation in order to identify enrichment of H3K4me3 and H3K27me3 marks in the promoter regions of selected HOX cluster genes, which show altered expression in cancers. HPV16 positive SiHa cell line served as a positive control and HPV negative C33A cell line served as a negative control for HPV16 E7 immunoprecipitation. The primers used for qPCR enrichment study are listed in Supplementary Table 9. The amplification plots and dissociation curves for the qPCR reactions are provided in Supplementary Figures 3 and 4, respectively. The primers targeting RPL-30 and EVX-1 promoters were used as positive control for H3K4me3 and H3K27me3 enrichment (Supplementary Figures 5 and 6), respectively, while ZNF333-3′ served as a negative control for H3K4me3 and H3K27me3 enrichment (Supplementary Figure 7). The H3K4me3 and H3K27me3 enrichment status in HPV16 positive cell line SiHa have been illustrated in Supplementary Figure 8.

### Statistical analysis

Kolmogorov-Smirnov test was used to check for the normality of the ΔCt values. Non-parametric test (Mann-Whitney U Test) was performed to check for statistical significance of the changes in expression levels, as the ΔCt values did not follow normal distribution. Multiple sets of experiments using cell line model were compared using ANOVA for statistically significant differences in expression levels between the experimental categories. All statistical analyses were done using SPSS v16.0. The p values were reported after performing Bonferroni multiple testing corrections.

## SUPPLEMENTARY FIGURES AND TABLES


