# Supplementary material for: Expression signatures of HOX cluster genes in cervical cancer pathogenesis: Impact of human papillomavirus type 16 oncoprotein E7
Source: Oncotarget. 2017 Mar 28;8(22):36591–602. doi: 10.18632/oncotarget.16619 (PMC5482679; doi:10.18632/oncotarget.16619)
Supplement: Supplementary file 1 [file oncotarget-08-36591-s001.pdf]

Expression signatures of HOX cluster genes in cervical cancer pathogenesis: Impact of human papillomavirus type 16 oncoprotein E7

SUPPLEMENTARY MATERIALS

SUPPLEMENTARY FIGURES AND TABLES

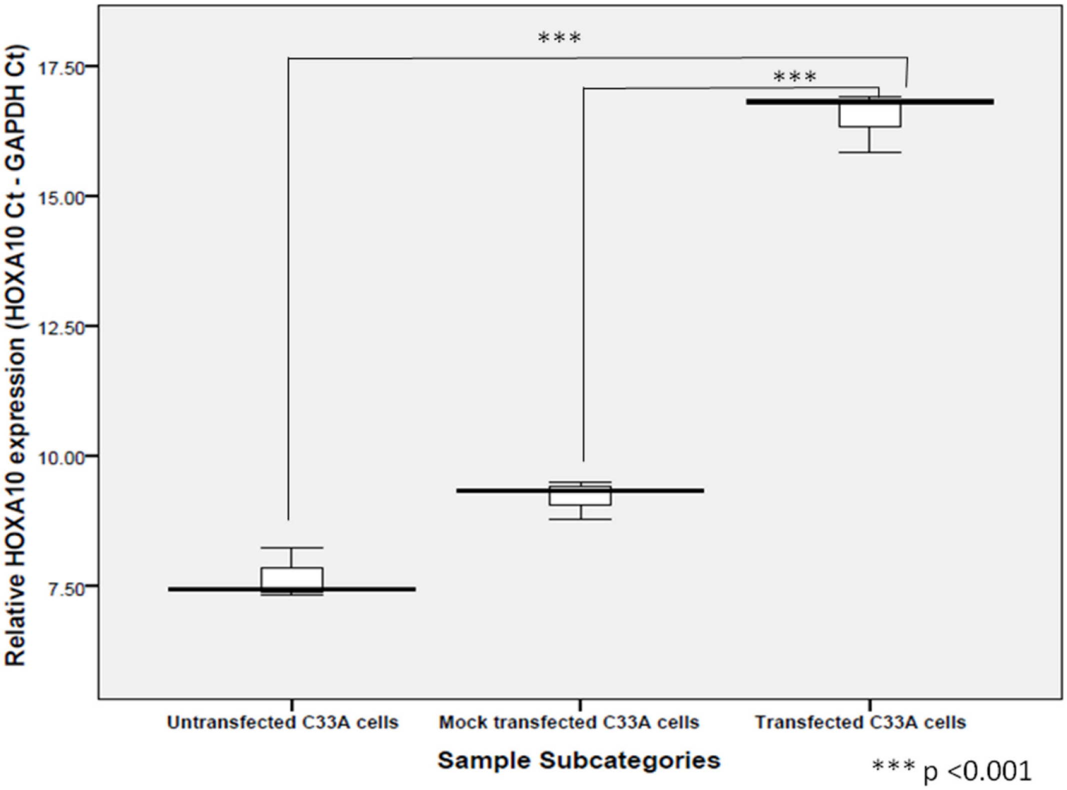

Supplementary Figure 1: Box plots representing expression of HOXA10, in C33A cells post-transfection of pcDNA3.1-HPV16 E7 vector in comparison to the untransfected and mock (empty vector) transfected C33A cells.

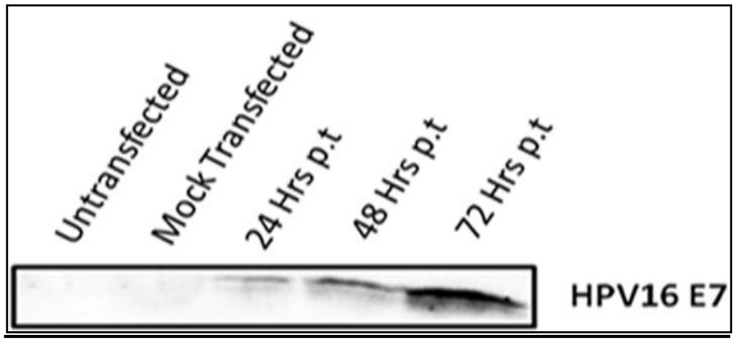

Supplementary Figure 2: Western blot showing the expression of HPV16 E7 at 24, 48 and 72 hrs post-transfection (p.t) of pcDNA3.1-HPV16 E7 vector [9].

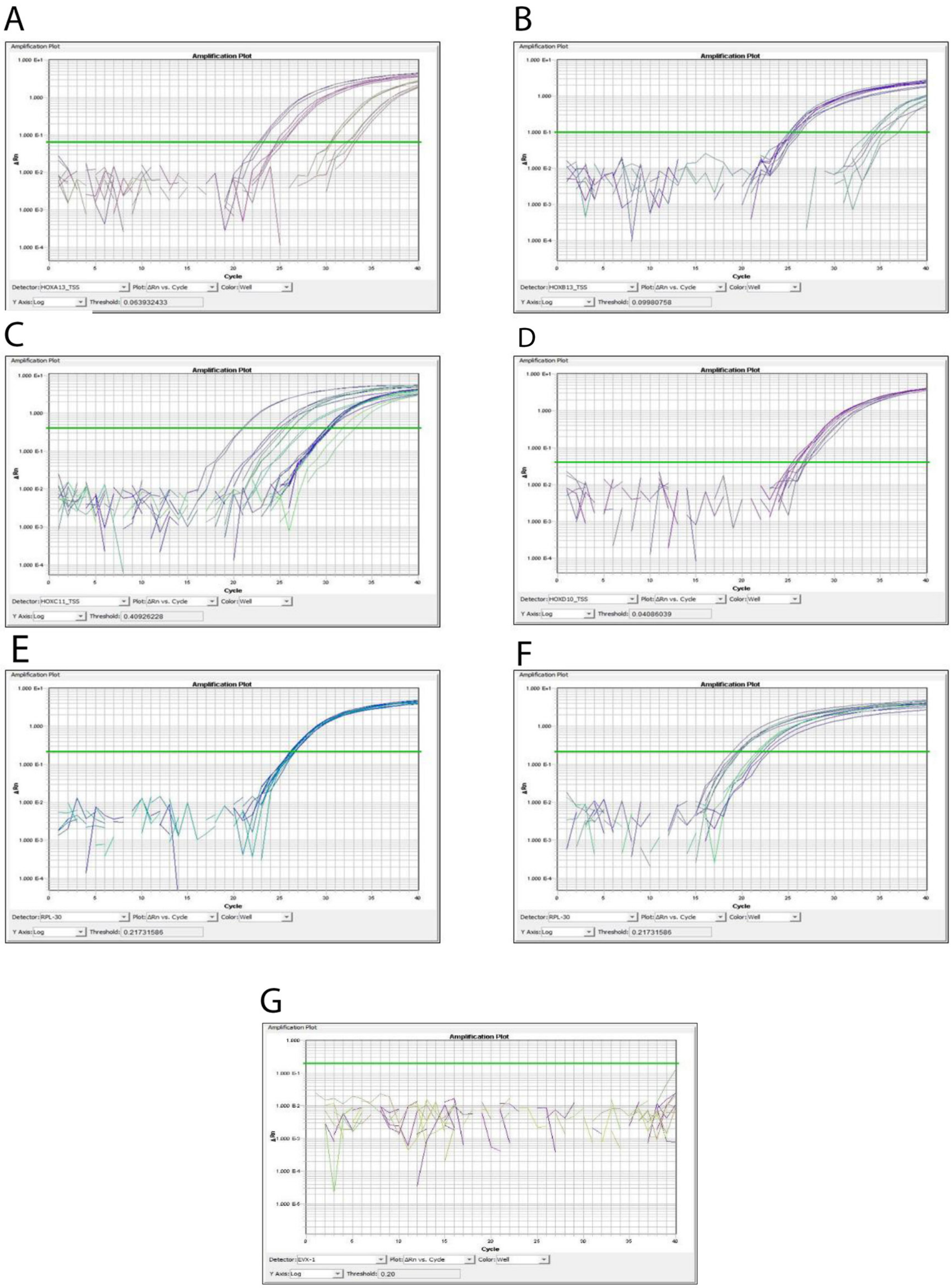

Supplementary Figure 3: Representative amplification plots of gene promoters under investigation (HOXA13, HOXB13, HOXC11, HOXD10, RPL30, EVX1 and ZNF333-3').

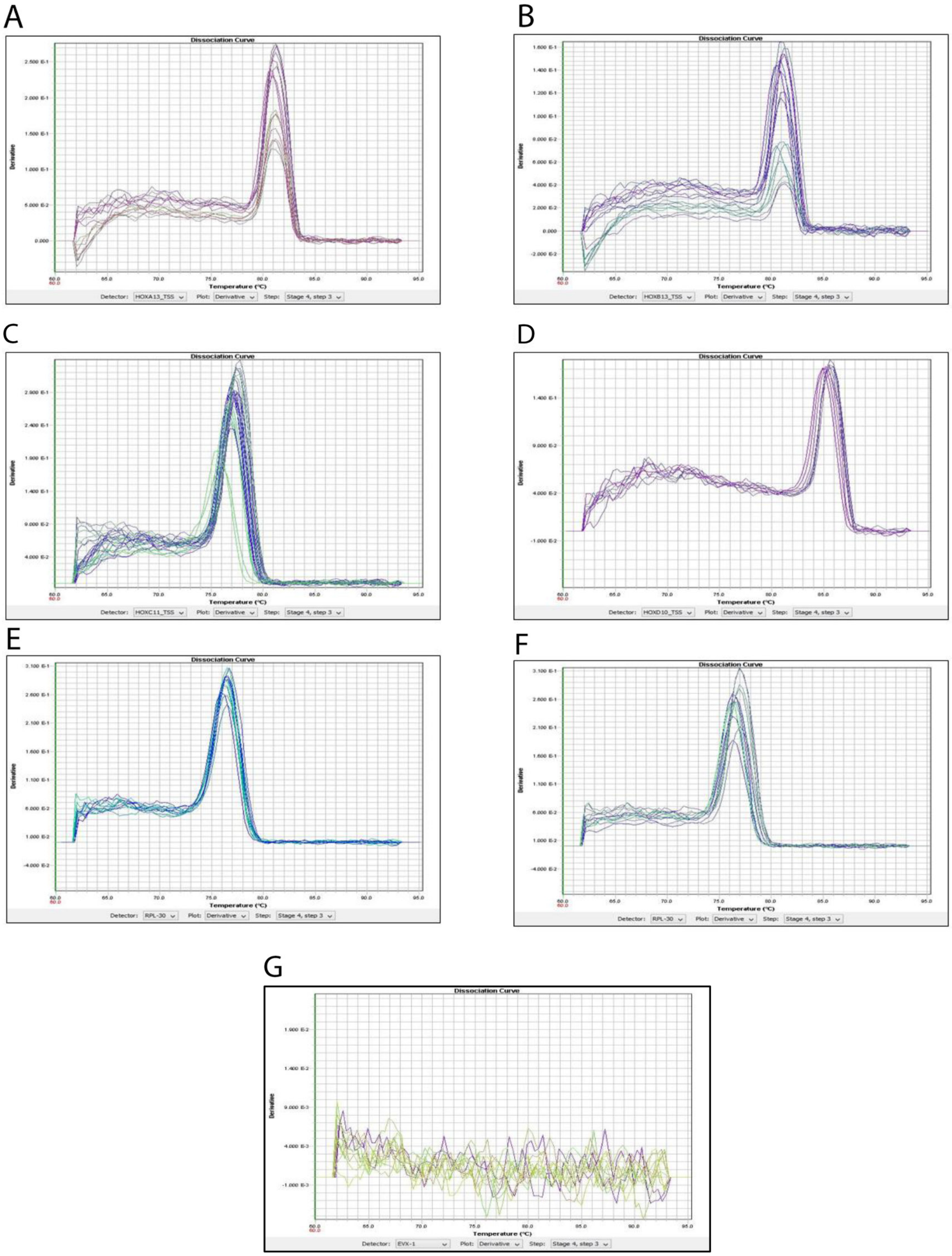

Supplementary Figure 4: Representative dissociation curves for checking the specificity of amplicons from the gene promoter regions under investigation (HOXA13, HOXB13, HOXC11, HOXD10, RPL30, EVX-1 and ZNF333-3').

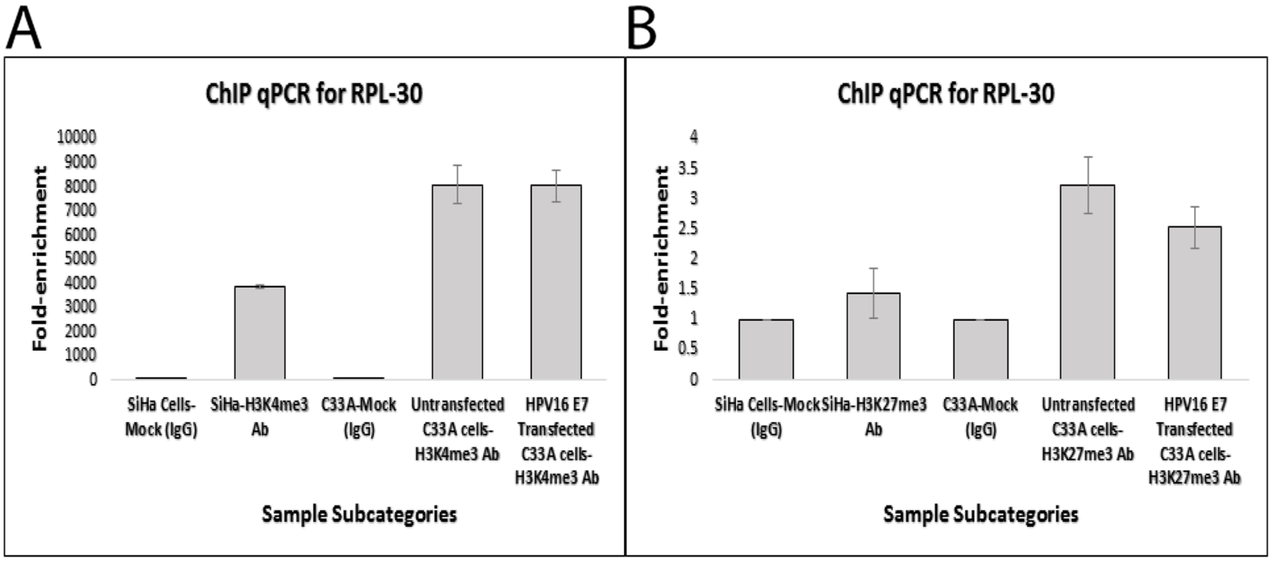

Supplementary Figure 5: q-PCR based analysis of enrichment of H3K4me3 and H3K27me3 marks at RPL30 promoter. **(A)** Significant enrichment of RPL30promoter for H3K4me3 occupancy as compared to IgG (control antibody) **(B)** Significant enrichment of RPL30 TSS for H3K27me3 occupancy as compared to IgG (control antibody).

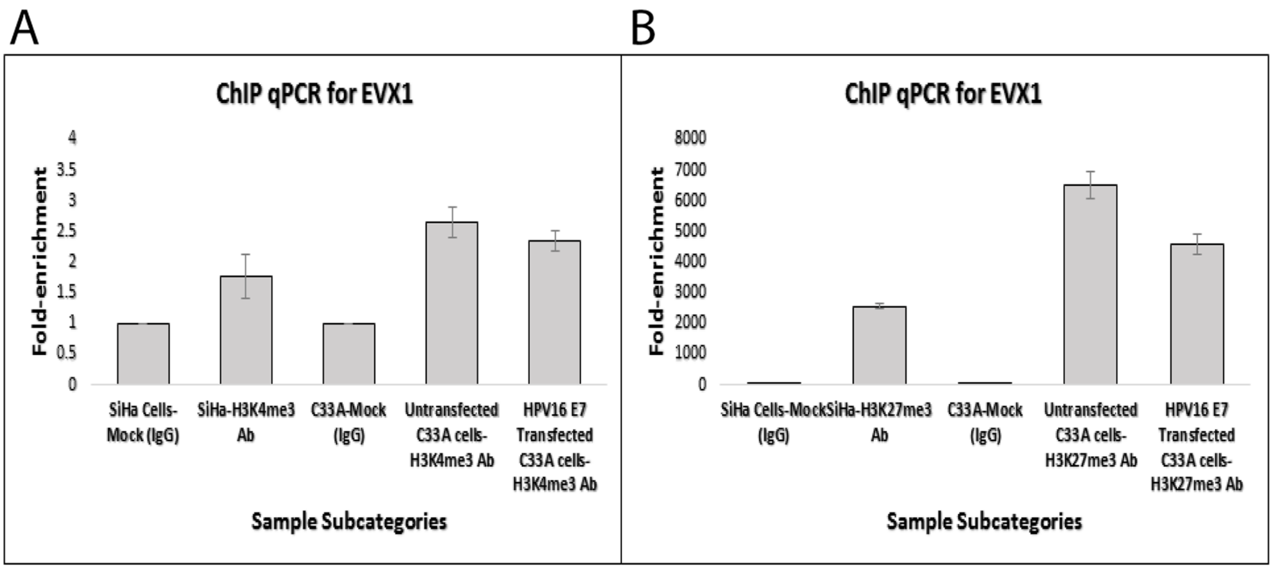

Supplementary Figure 6: q-PCR based analysis of enrichment of H3K4me3 and H3K27me3 marks at EVX1 promoter. **(A)** Significant enrichment of EVX1 promoter for H3K4me3 occupancy as compared to IgG (control antibody) **(B)** Significant enrichment of EVX1 TSS for H3K27me3 occupancy as compared to IgG (control antibody).

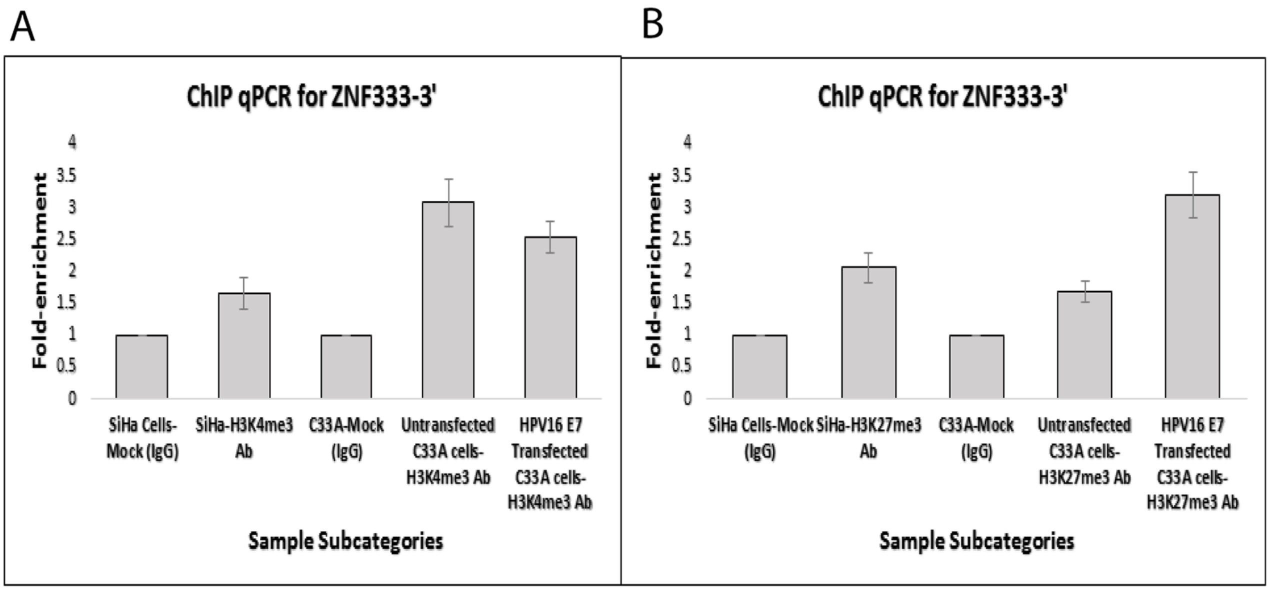

Supplementary Figure 7: q-PCR based analysis of enrichment of H3K4me3 and H3K27me3 marks at ZNF333-3'. **(A)** Significant enrichment of ZNF333-3' for H3K4me3 occupancy as compared to IgG (control antibody) **(B)** Significant enrichment of ZNF333-3' for H3K27me3 occupancy as compared to IgG (control antibody).

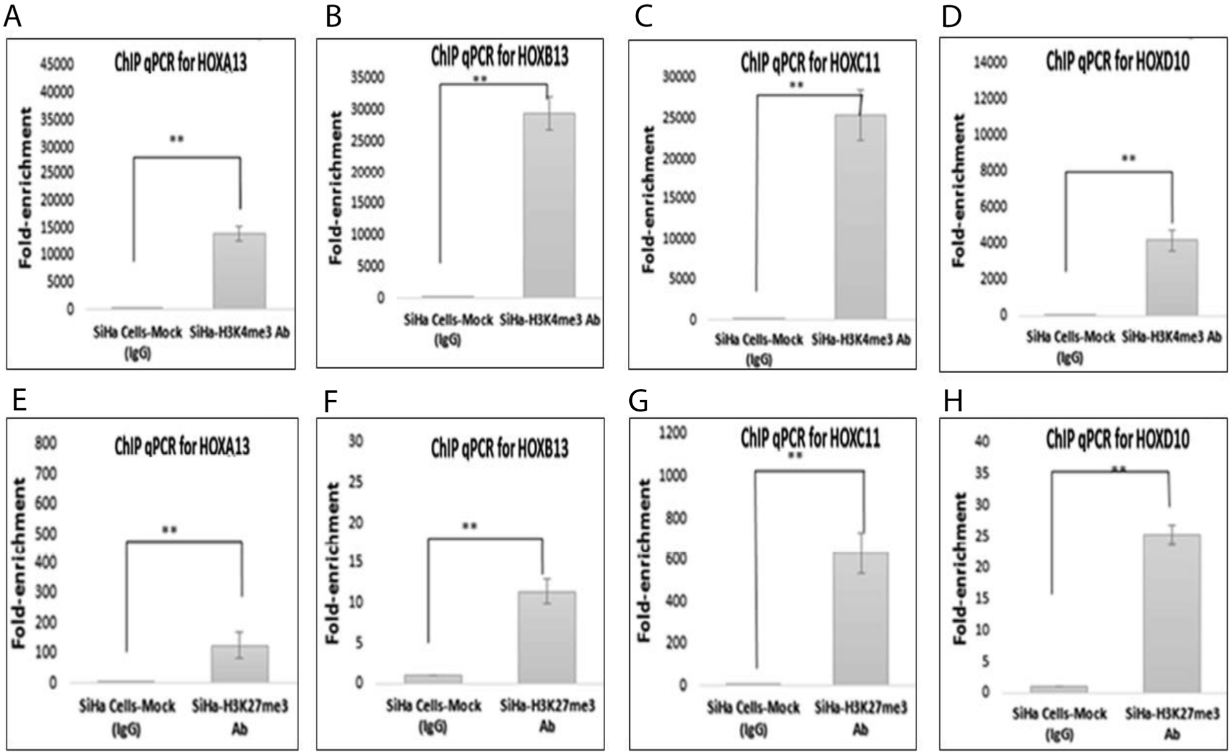

Supplementary Figure 8: q-PCR based analysis of enrichment of H3K4me3 and H3K27me3 marks at the promoters of HOX cluster genes in HPV16 positive SiHa cells. Fold-enrichment of H3K4me3 marks at **(A)** HOXA13, **(B)** HOXB13, **(C)** HOXC11, **(D)** HOXD10 promoters, Fold-enrichment of H3K27me3 marks at **(E)** HOXA13, **(F)** HOXB13, **(G)** HOXC11, **(H)** HOXD10 promoters.

Supplementary Table 1: Expression status of HOX cluster genes among the CaCx cases as identified by microarray analysis

HOX Cluster	HOX Cluster members	Expression status among HPV16 positive CaCx cases as compared to histopathologically normal controls (Fold-change; p-value)
**HOXA**	HOXA5	3.59-fold; p=0.016 (Up-regulation)
	HOXA10	2.88-fold; p<0.001 (Down-regulation)
	HOXA13	2.59-fold; p<0.001 (Up-regulation)
**HOXB**	HOXB13	2.14-fold; p=0.002 (Up-regulation)
**HOXC**	HOXC4	7.63-fold; p<0.001 (Up-regulation)
	HOXC8	8.74-fold; p<0.001 (Up-regulation)
	HOXC9	7.27-fold; p<0.001 (Up-regulation)
	HOXC11	2.38-fold; p=0.003 (Up-regulation)
**HOXD**	HOXD1	3.02-fold; p<0.001 (Up-regulation)
	HOXD4	2.64-fold; p=0.018 (Up-regulation)
	HOXD10	5.62-fold; p<0.001 (Up-regulation)
	HOXD11	2.84-fold; p=0.035 (Up-regulation)

Supplementary Table 2: Expression status of HOX cluster genes among the CaCx cases as confirmed by qRT-PCR

HOX Cluster	HOX Cluster members analysed	Expression status among HPV16 positive CaCx cases as compared to HPV negative controls (Fold-change, p-value)
**HOXA**	HOXA10	8.92-fold; p=0.035 (Down-regulation)
	HOXA13	10.32-fold; p=0.014 (Up-regulation)
**HOXB**	HOXB13	6.98-fold; p=0.038 (Up-regulation)
**HOXC**	HOXC8	32.8-fold; p=0.0014(Up-regulation)
	HOXC9	26.76-fold; p= 0.0014(Up-regulation)
	HOXC11	12.46-fold; p=0.035 (Up-regulation)
**HOXD**	HOXD10	27.5-fold; 0.021 (Up-regulation)

Supplementary Table 3: Correlation analysis between expression levels of HOX cluster transcripts and E-Cadherin

Transcript name	Pearson's correlation coefficient	p-value
**HOXA10**	0.645	0.00175*
**HOXA13**	-0.036	0.98
**HOXB13**	-0.467	0.0014*
**HOXC8**	-0.134	0.5495
**HOXC9**	-0.003	0.98
**HOXC11**	-0.122	0.5495
**HOXD10**	0.162	0.98

* Statistically significant

Supplementary Table 4: Correlation analysis between expression levels of HOX cluster transcripts and Vimentin

Transcript name	Pearson's correlation coefficient	p-value
**HOXA10**	-0.456	0.014*
**HOXA13**	0.161	0.32
**HOXB13**	0.328	0.035*
**HOXC8**	0.013	0.914
**HOXC9**	-0.027	0.914
**HOXC11**	-0.053	0.914
**HOXD10**	-0.165	0.32

* Statistically significant

Supplementary Table 5: Correlation analysis between expression levels of HOX cluster transcripts and HPV16 E7 using microarray expression values

See Supplementary File 1

Supplementary Table 6: Expression status of HOX cluster genes in C33A cells with and without HPV16 E7 expression

HOX Cluster	Expression status among C33A cells as compared to Untransfected C33A cells (Fold-change, p-value)	Expression status among C33A cells as compared to Mock (Empty Vector) transfected C33A cells (Fold-change, p-value)
**HOXA13**	309.68-fold; p<0.001 (up-regulation)	165.79-fold; p<0.001 (up-regulation)
**HOXB13**	87.02-fold; p<0.001 (Up-regulation)	42.69-fold; p<0.001 (Up-regulation)
**HOXC11**	67.48-fold; p<0.001(Up-regulation)	38.6-fold; p<0.001(Up-regulation)
**HOXD10**	88.58-fold; p<0.001 (Up-regulation)	66.02-fold; p<0.001 (Up-regulation)

Supplementary Table 7: Mean Fold-enrichment of H3K4me3 gene activation marks and H3K27me3 gene repressive marks at the promoters of four HOX cluster genes.

See Supplementary File 2

Supplementary Table 8: Primer Sequences for qRT-PCR (SYBR green assay) based expression analysis.

See Supplementary File 3

Supplementary Table 9: Primer Sequences for ChIP-qPCR.

See Supplementary File 4
